# Sucrose but not arsenic induce hepatic steatosis which correlates with calpain-1 inhibition

**DOI:** 10.1371/journal.pone.0339586

**Published:** 2025-12-30

**Authors:** Myrian Velasco, Rosa Isela Ortiz-Huidobro, Ana María Salazar, Marcia Hiriart, Patricia Ostrosky-Wegman, Pablo Pánico

**Affiliations:** 1 Department of Genomic Medicine and Environmental Toxicology, Instituto de Investigaciones Biomédicas, Universidad Nacional Autónoma de México, Mexico City, Mexico; 2 Department of Cognitive Neurosciences, Instituto de Fisiología Celular, Universidad Nacional Autónoma de México, Mexico City, Mexico; University of Louisville, UNITED STATES OF AMERICA

## Abstract

Exposure to arsenic or sugary drinks can increase the risk of developing metabolic dysfunction-associated steatotic liver disease (MASLD), although the mechanisms involved and its possible interactions remain unexplored. This work aimed to characterize the development of MASLD and their molecular mechanisms, induced in male Wistar rats consuming 20% sucrose (S), 50 ppm sodium arsenite (A), or both (A + S) through drinking water for 2 months. We found that rats consuming S developed liver steatosis and extensive fibrosis. Liver steatosis induced by S and A + S correlated with decreased calpain-1 protein abundance and calpain-dependent αII-spectrin proteolysis. We showed that calpain-1 inhibition *in vitro* induces lipid accumulation in hepatocytes probably by increasing PPARγ protein levels in the hepatic HepG2 cell line. Concordantly, PPARγ protein levels were increased in the livers of S-treated rats. A-treated rats did not develop steatosis, displayed decreased calpain-1 levels but its proteolytic activity remained intact. Treatment with A + S induced liver steatosis but displayed less fibrosis than S-treated rats. Although A + S reduced calpain-1 levels and activity, the levels of PPARγ were not induced in these rats. Our results suggest that liver steatosis induced by sucrose could be related with impaired calpain activity, which could promote PPARγ increase. However, some of these alterations induced by sucrose are attenuated by co-administering arsenic, suggesting that there are other calpain-dependent mechanisms involved in liver steatosis. The graphical abstract was generated using BioRender (License in S5 File).

## 1. Introduction

Metabolic dysfunction-associated steatotic liver disease (MASLD) is a condition characterized by the accumulation of lipids in the liver greater than 5% of the total liver weight in the absence of excessive alcohol consumption [1]. This condition is the most common chronic liver disease worldwide; it is the second leading cause of liver transplant and is one of the most important predisposing factors to develop cirrhosis and hepatocellular carcinoma [1]. MASLD also increases the risk of developing dyslipidemias, atherosclerosis, and hypertension [1].

The first stage of MASLD begins with an imbalance in fatty acid metabolism in the liver due to a higher rate of *de novo* lipogenesis and uptake of circulating free fatty acids, along with a decreased fatty acid oxidation and triglyceride export, leading to hepatic steatosis [2]. The second stage occurs when the excess of lipids in hepatocytes triggers oxidative stress and inflammation, promoting the development of non-alcoholic steatohepatitis [2]. Finally, when these processes are not controlled, they can promote the development of liver fibrosis and hepatocellular carcinoma [3].

The development of MASLD is influenced by multiple risk factors, including sugary drinks and exposure to environmental contaminants with endocrine disruptor activity, such as arsenic [1,4,5]. Although chronic treatment with sucrose for six months or trivalent arsenic for three months promotes liver steatosis in rats [6,7], the specific alterations and pathways involved in the early stages of MASLD induced by these factors are not characterized. Moreover, this interaction could determine the heterogeneity of MASLD physiopathology and limit therapeutic options [3]. Thus, it is relevant to characterize whether sugary drinks and arsenic ingestion interact on signaling pathways and physiopathological processes involved in the early stages of MASLD development.

At the molecular level, it is proposed that deregulation of the calpain system contributes to the progression of MASLD. The calpain system consists of a family of 15 non-lysosomal cysteine proteases, along with the small regulatory subunit of calpain-1 (CAPN1) and calpain-2 (CAPN2) called CAPNS1 and the inhibitory protein calpastatin (CAST) [8]. In the liver endothelium, calpains promote excessive protein degradation, which increases the release of branched-chain amino acids and contribute to lipid biosynthesis in hepatocytes [9]. Additionally, lipid peroxidation products induce the overexpression of CAPN1 in the liver, promoting increased lysosomal permeability, the release of lysosomal proteases into the cytosol, and hepatocyte cell death [10]. However, the roles of these proteases in the development of MASLD induced by sucrose and arsenic have not been characterized.

In our previous work, we found that the consumption of sucrose and sodium arsenite for eight weeks interact in complex ways to promote the development of the signs of metabolic syndrome in male Wistar rats [11]. Notably, we showed that 1) arsenic did not influence body weight gain induced by sucrose, 2) sucrose, arsenic, and the combination of both produced systemic and muscle-specific insulin resistance without additive effects, mainly due to differences in the signaling pathways altered by each treatment, and 3) sucrose increased plasma triglyceride levels, while arsenic prevented this increase [11]. Since the liver is one of the main organs involved in the metabolic regulation of the body, and both sugary drinks and arsenic are known risk factors for developing MASLD, we aimed to characterize the development of MASLD and the involvement of the calpain system in this pathology in the liver of male Wistar rats consuming sucrose and arsenic in water for eight weeks. This study was focused on males because the development of MASLD is heavily influenced by sex, and its prevalence is higher in males than in pre-menopausal females [12].

## 2. Methods

### 2.1. Animals and treatments

According to the principles for reducing the number of animals used for research, we performed the experiments for this work on plasma and liver samples obtained from the same rat cohort from our previous work [11]. All animal experiments were approved by the Bioethics Committee of the Institute of Cellular Physiology, UNAM (UNAM, CICUAL MHU189−22) and animal care was performed according to the International Guiding Principles for Biomedical Research Involving Animals, Council for International Organizations of Medical Sciences, 2010. Myrian Velasco received special training from the staff of the animal facility of the Instituto de Fisiología Celular, UNAM to take care of the study animals

A total of 24 male Wistar rats (two-month-old, 250–280 g body weight) were randomly assigned to each treatment group: 20% sucrose (Food grade, Great Value), 50 ppm sodium arsenite (Sigma Aldrich, CAS 7784-46-5, Purity 99%), or both treatments in drinking water for 8 weeks, and fed with standard rodent diet (LabDiet 5001) at the vivarium of the Institute of Cellular Physiology, UNAM (12 h light/dark cycle, 20–23 °C, and 40% relative humidity). Arsenic dose was based on previous reports showing that arsenite toxicokinetic differ between rat and human, trivalent arsenicals bind to the thiol groups in rat hemoglobin, reducing its free plasma concentration [13]. Human endpoints were not used for this study because we previously showed that the dose of arsenic ingested in our model ranges between 4 ang 5 mg/ kg of body weight/ day, which is below the non-adverse effect level (NOAEL) for repeated doses in rats, minimizing the risk of acute toxic effects that could compromise animal welfare [11]. Drinking water with the treatments was replaced every three days to avoid microbial growth and arsenic oxidation. No animal died before the end of the treatments, and their welfare was monitored every week, quantitating body weight gain, and food and water intake [11].

At the end of the eighth week of treatment, the rats were sacrificed with an overdose of sodium pentobarbital (40 mg/kg) after overnight fasting (6 animals in each treatment) to obtain the tissues, including samples from the right ventral lobe of the liver. Samples were stored at −70 °C until they were processed. The quantitative measurements of alanine aminotransferase (ALT, Cat# 318−10) and aspartate aminotransferase (AST, Cat# 319−10) in plasma were performed in the same animals that were reported in our previous work (20 animals for each treatment) using enzymatic methods according to the manufacturer´s instructions (Sekisui Diagnostics).

### 2.2. Histology

Liver sections were embedded in Tissue-Tek O.C.T. mounting medium and quickly frozen in isopentane on liquid nitrogen. Histological sections with a thickness of 3 µm were prepared, fixed with 4% paraformaldehyde, and stained with oil red O (ORO) or hematoxylin & eosin. Independently, liver sections were fixed with 4% paraformaldehyde and embedded in paraffin to obtain histological sections of 7 µm for Masson’s trichrome staining. Images were obtained with a DM500 compound microscope (Leica Microsystems), using the 10 X, 40 X and 100 X objectives. The percentage of the area stained with ORO was measured using the Fiji software [14].

### 2.3. Cell culture and *in vitro* treatments

The hepatocarcinoma HepG2 cell line was obtained from the American Type Culture Collection (ATCC) and maintained according to their protocols. The cells were cultured in Eagle’s minimum essential medium (EMEM, ATCC) supplemented with 10% fetal bovine serum (Gibco) and kept in a humidified incubator at 37 °C and 5% CO_2_. For the experiments, the cells were seeded at a density of 0.1 X 10^6^ cells/cm^2^, as this density results in a 70–80% confluence by the end of the treatment. The cells were treated 24 h after seeding with 10, 50 and 100 µM of calpain inhibitor XII (Santa Cruz Biotechnology), equimolar concentrations of glucose and fructose (15 and 25 mM) or 1 µM sodium arsenite for 72 h. Control cells were treated with vehicle (dimethyl sulfoxide).

### 2.4. Oil red O staining

After the treatment with the calpain inhibitor, the cells were washed with PBS twice and fixed with 4% paraformaldehyde for one hour. Then, the cells were stained for 30 min with a 0.3% oil red O solution in 60% isopropanol. Cells were washed 5 times with distilled water and dried at room temperature. Subsequently, the stain was extracted with absolute isopropanol for 20 min with agitation. The absorbance of the supernatant was quantified at 515 nm using an ELISA reader. Each experiment was performed with three independent wells for each condition.

### 2.5. Determination of cell viability

After the treatments, the culture medium was removed, and the cells were incubated in 0.016 mg/mL fluorescein diacetate dissolved in serum-free medium for 5 min at room temperature with low agitation. Cells were washed once with PBS and the fluorescence levels were measured with a Fluoroskan Ascent (Thermo) with excitation/emission pair of 488/533 nm. Each experiment was performed with three independent wells for each condition. Puromycin (2 µg/mL) was used as a positive control of cell death.

### 2.6. Quantitative western blot

Total protein was extracted and quantitative immunoblot conditions were performed essentially as previously described [11]. Briefly, total protein was extracted with RIPA buffer, supplemented with cOmplete™, Mini Protease Inhibitor Cocktail (Roche), and 1 mM sodium fluoride. The samples were homogenized, sonicated and centrifuged to discard tissue debris. The protein concentration was measured with the DC™ Protein Assay kit (Bio-Rad). Equal amounts of each protein sample were denatured with Laemmli buffer at 85 °C for 5 min. The samples were run on 12% SDS-polyacrylamide gels, except for αII spectrin, which was evaluated on 8% SDS-polyacrylamide gels. Proteins were transferred to PVDF membranes in a semi-dry chamber (BioRad) at 20 V for 50 min. Membranes were blocked with tris buffered saline with 0.1% Tween 20 (TBS-T) and 4% low-fat milk for Western blot (Santa Cruz Biotechnology).

The primary antibodies were incubated overnight at 4 °C: rabbit anti-calpain-1 (#2556, 1:1000, Cell Signaling), rabbit anti-calpain-2 (ab126600, 1:1000, Abcam), rabbit anti-calpain small subunit 1 (A307324, 1:1000, antibodies.com), rabbit anti-calpastatin (ab28252, 1:1000, Abcam), rabbit anti-calpain-10 (ab28226, 1:1000, Abcam), rabbit anti-αII spectrin (PA5–35383, 1:1000, Invitrogen), rabbit anti-PPARγ (PA3-821A, 1:1000, Invitrogen). The secondary antibody used was the Peroxidase IgG Fraction Monoclonal Mouse Anti-Rabbit IgG, light chain speciﬁc (211-032-171, 1:6000, Jackson Immunoresearch). The signal was detected with the Enhanced Chemiluminescence Prime kit (Amersham) in a Li-Cor C-Digit scanner (Li-Cor Biosciences). The optical density of the bands was determined using the Image Studio Lite Ver. 5.2 software (Li-Cor Biosciences) (The original images from the gels are available in S2 File).

### 2.7. RNA extraction and quantitative RT-PCR

Total RNA was extracted from 20 mg of liver with the RNeasy Mini kit (Qiagen), according to the manufacturer’s protocol. The concentration of the RNA samples was determined using a Nanodrop spectrophotometer (Thermo Scientific). The cDNA was synthesized from 500 ng of total RNA using the SuperScript III First-Strand Synthesis SuperMix kit (Invitrogen) and random hexamers, according to manufacturer’s instructions. Taqman probes (Applied Biosystems) against the mRNA of CAPN1 (Rn01479699_m1), CAPN2 (Rn00567422_m1), CAPNS1 (Rn01498486_g1), CAPN10 (Rn00581535_m1), and CAST (Rn00583952_m1) were used to evaluate the expression levels by quantitative real-time PCR, using 15 ng of cDNA. The expression levels of the glucuronidase B (GUSB, Rn00566655_m1) gene were used as the housekeeping control. The analysis of the expression levels was performed according to the 2^-ΔΔCt^ method [15].

### 2.8. Statistical analysis and image assembly

All determinations in rat livers were done in at least four animals for each treatment. We performed at least three independent experiments for the *in vitro* experiments in HepG2 cells. The specific sample number for each experiment is specified in the figure legends. The analysis of the data was performed with GraphPad Prism 8.0 software. Data was assessed for normal distribution with Shapiro-Wilk test. For the *in vivo* model, we performed two-way ANOVA with Tukey’s post hoc test, while for the *in vitro* model we used one-way ANOVA and Tukey’s post hoc test. Differences were considered statistically significant at p < 0.05 (Raw data and statistical analysis results are available in S3 Table and S4 Table). Graphs present the mean ± standard deviation (SD), and each circle denotes the individual data points. The ﬁnal ﬁgures were assembled using Adobe Photoshop 2022.

## 3. Results

**Fig 1:**
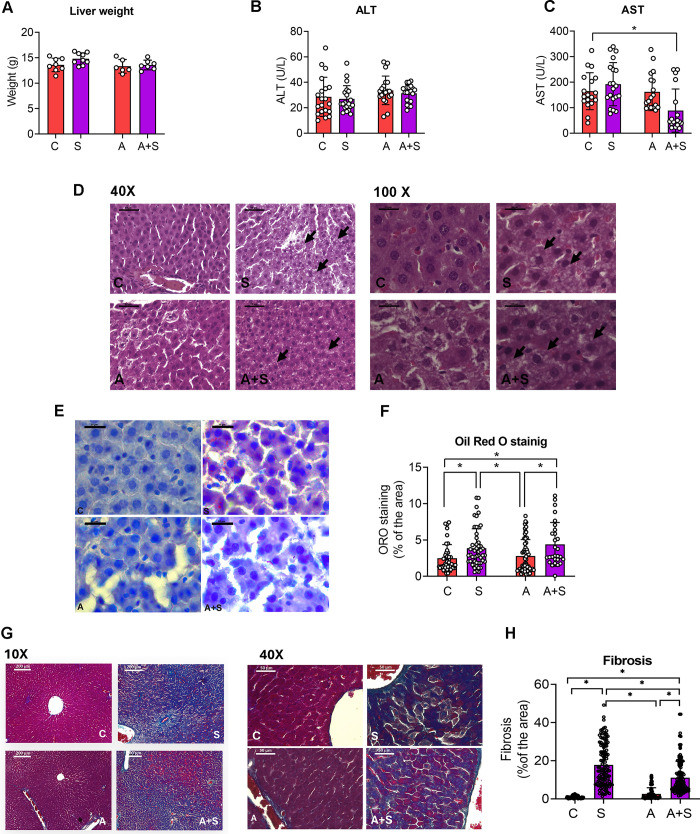
Sucrose and arsenic + sucrose induce hepatic steatosis and fibrosis. Male Wistar rats were treated with 20% sucrose (S), 50 ppm of sodium arsenite (A) or both (A + S) through drinking water for 8 weeks. A) Total liver weight. B) Plasma levels of alanine transaminase (ALT) reported as units/liter. C) Plasma levels of aspartate transaminase (AST) reported as units/liter. D) Representative images of hematoxylin and eosin staining. Arrows show ballooned hepatocytes. Left scale bars = 50 μm, right scale bars = 20 μm. E) Representative images of Oil Red O staining in liver, scale bars = 20 μm. F) Percentage area of tissue positive for Oil Red O staining. G) Representative images of liver stained with Masson’s trichrome staining. Left scale bars = 200 μm, right scale bars = 50 μm. H) Percentage area of tissue positive for fibrosis. In each graph, bars denote the mean ± SD, with independent animals presented as dots, except in F and H, where each point represents the microscope fields quantified from at least 3 independent animals per condition. Analysis by two-way ANOVA with Tukey’s post-hoc test. * denotes statistically significant differences in the post-hoc test with p < 0.05.

Our previous work demonstrated that exposure through drinking water with 50 ppm of arsenic did not affect weight gain induced by 20% sucrose in male rats. Moreover, we demonstrated that sucrose (S), arsenic (A) and exposure to both factors (A + S) induced peripheral insulin resistance, while arsenic prevented the increase in plasma triglycerides induced by sucrose consumption [11]. In this study, we observed that none of the treatments altered the liver weight and the alanine transaminase (ALT) levels in plasma (Fig 1A and 1B). In animals treated with A + S there was a statistically significant decrease in the levels of aspartate transaminase (AST) in plasma compared to controls (C) (Fig 1C).

Histological analysis showed that sucrose promotes the development of ballooned hepatocytes, independently of arsenic co-exposure (Fig 1D). Concordantly with the presence of ballooned hepatocytes, the livers from animals treated with S and A + S had increased accumulation of lipids, compared with C and A groups. Although there was no additive effect between arsenic and sucrose on lipid accumulation, the morphological characteristics of these lipid deposits differed between S and A + S livers. While the livers from S-treated animals displayed big lipid droplets, the lipid accumulation in A + S-treated animals was evenly distributed throughout hepatocytes’ cytoplasm (Fig 1E and 1F).

We also detected that the livers of animals treated with S and A + S developed extensive areas of fibrosis, compared with C and A. However, the extension of fibrotic areas was statistically significant higher in animals exposed to S than those exposed to A + S (Fig 1G and 1H).

**Fig 2:**
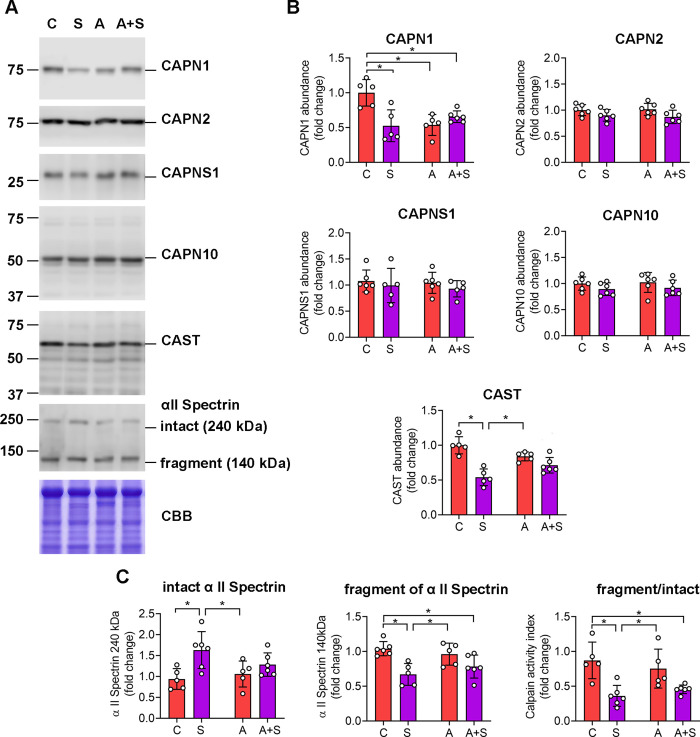
Exposure to sucrose and arsenic + sucrose impairs CAPN1 activity in the liver. A) Representative Western blot of CAPN1, CAPN2, CAPNS1, CAPN10, CAST, and αII-spectrin in liver samples from male Wistar rats treated with 20% sucrose (S), 50 ppm of sodium arsenite (A) or both (A + S) through drinking water for 8 weeks. Coomassie brilliant blue (CBB) staining was used as a loading control. B) Quantification of the protein abundance of the members of the calpain system. C) Quantification of the intact αII-spectrin (240 kDa), the fragment generated by calpain activity (140 kDa) and the ratio of the abundance of the fragment/ intact αII-spectrin. The optical density of the bands was normalized to the optical density of the entire CBB lane. Data are presented as fold change relative to the mean value of the control animals. The bars denote the mean ± SD of at least 5 animals per condition. Each animal is represented by dots. Analysis by two-way ANOVA with Tukey’s post-hoc test. * denotes statistically significant differences in the post-hoc test with p < 0.05.

Next, we evaluated potential alterations in the protein levels of CAPN1, CAPN2, CAPN10, CAPNS1, and their endogenous inhibitor, CAST in the liver. The analysis showed neither treatment altered the protein levels of CAPN2, CAPN10 and CAPNS1 (Fig 2A and 2B). Of note, unlike our results in other tissues that express several CAPN10 isoforms [11,16], we only found one isoform of CAPN10 in the liver of about 60 kDa (Fig 2A). Contrarily, S, A, and, A + S treatments decreased CAPN1 protein levels compared with C (Fig 2A and 2B). There was a reduction in the protein levels of CAST in animals consuming S, but this effect was not statistically significant in animals consuming A + S compared with C, and A (Fig 2A and 2B).

Calpain activity *in vivo* can be monitored by assessing the proteolysis of their targets, and αII-spectrin is one of the best-characterized targets of CAPN1 and CAPN2 [17]. This protein is part of the cell membrane cytoskeleton and is primarily cleaved by CAPN1 under physiological and pathological conditions, generating a 140 kDa fragment [18]. In the livers of the animals consuming S there was an increase in the levels of the intact αII-spectrin band (250 kDa), while this effect was lost in animals consuming A + S compared with C, and A (Fig 2A and 2C). Conversely, S, and A + S reduced the abundance of the 140 kDa fragment compared with C, and A (Fig 2A and 2C). We calculated a ratio of the 140 kDa fragment to intact αII-spectrin to assess the calpain activity in treated animals’ livers. The S and A + S treated animals had a decrease in this ratio, compared with A and C, which indicates that these treatments reduce calpain activity in the liver (Fig 2A and 2C).

**Fig 3:**
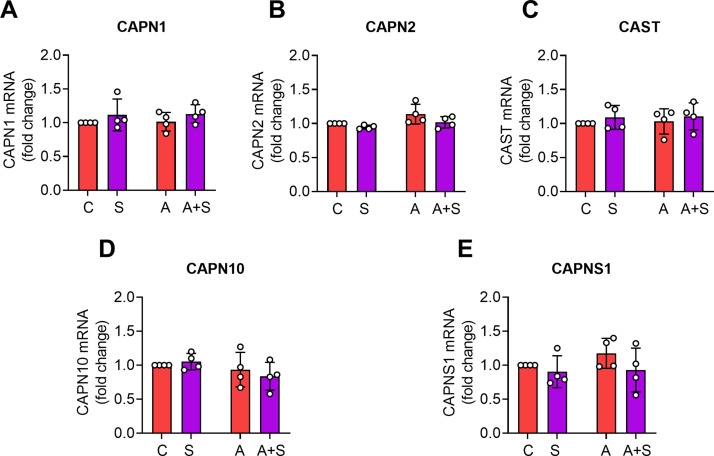
The changes in calpain system protein abundance and activity were not related to changes in mRNA expression. The mRNA expression of A) CAPN1 mRNA. B) CAPN2 mRNA. C) CAST mRNA. D) CAPN10 mRNA. E) CAPNS1 were quantitated by real-time PCR and normalized to the expression levels of glucuronidase B (GUSB). Data are reported as fold change relative to control animals. The bars denote the mean ± SD of at least 4 animals per condition; each point represents an independent animal. Analysis by two-way ANOVA with Tukey’s post-hoc test.

We further evaluated whether the changes in the protein abundance of the calpain system members were related to alterations in mRNA expression. However, we did not observe differences between treatments (Fig 3).

**Fig 4:**
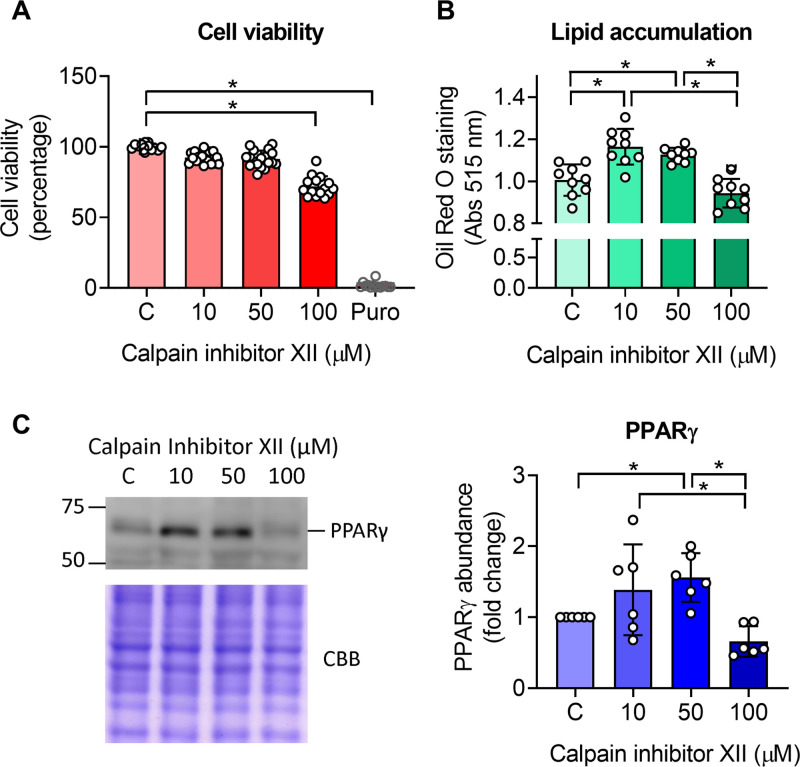
Pharmacological inhibition with a CAPN1-selective inhibitor (calpain inhibitor XII) promotes steatosis and PPARγ upregulation in hepatocarcinoma HepG2 cells. A) Cell viability of HepG2 cells treated with calpain inhibitor XII for 72 h. Puro: positive control of cells treated with puromycin. B) Lipid accumulation in HepG2 cells treated with calpain inhibitor XII for 72 h. Data are expressed as the absorbance of ORO staining at 515 nm and normalized to protein content in the well. C) Representative Western blot and quantification of the abundance of PPARγ in HepG2 cells treated with calpain inhibitor XII. In A and B the bars denote the mean ± SD of 3 independent experiments with technical triplicates. In C the bars denote the mean ± SD of 5 independent experiments. Each point represents each well evaluated. Analysis by one-way ANOVA with Tukey’s post-hoc test. * denotes statistically significant differences in the post-hoc test with p < 0.05.

Since no reports associate the development of hepatic steatosis with a decrease in calpain activity and CAPN1 protein abundance, we performed a prove of concept experiment in the human hepatocarcinoma cell line model (HepG2). These cells were treated for 72 h with 10, 50, and 100 µM of calpain inhibitor XII, which is a selective inhibitor for CAPN1 [19]. We only observed a decrease in cell viability with the highest concentration of the inhibitor (Fig 4A). Intracellular lipid accumulation increased after the treatment with the non-cytotoxic concentrations of 10 and 50 µM of the calpain inhibitor (Fig 4B). Thus, suggesting that calpain inhibition can promote lipid accumulation in hepatocytes.

To explore the possible mechanism by which calpain inhibition promotes hepatic steatosis, we evaluated the protein abundance of PPARγ, which is cleaved by CAPN1 in cell-free systems [20] and promotes de novo lipogenesis in MASLD [21]. In the HepG2 cells, treatment with 10 and 50 µM of calpain inhibitor XII resulted in increased PPARγ protein abundance. In comparison, the abundance of this protein decreased after treatment with 100 µM of the inhibitor (Fig 4C).

We further explored the correlation between CAPN1 and PPARγ induction in the in vitro system by exposing the cells to equimolar concentrations of glucose and fructose, which are the carbohydrates (CHO) absorbed in the gut after sucrose is broken down by sucrase [22], and arsenic, in the presence or absence of calpain inhibitor XII (S1 Fig). We observed that arsenic reduced cell viability, but this effect was rescued by CHO co-exposure. However, the improvement in cell viability induced by CHO was absent in the presence of the calpain inhibitor (S1 Fig). Lipid accumulation was induced by the calpain inhibitor, and it was not influenced by CHO nor by arsenic exposure (S1B Fig). These data agree with previous works showing that HepG2 do not develop steatosis induced by carbohydrates [23,24]. PPARγ protein levels increased in the presence of 25 mM of CHO and by arsenic, but this increased was blunted in cells co-treated with CHO and arsenic. Interestingly, these effects on PPARγ were not observed when the calpain inhibitor was present (S1C Fig).

**Fig 5:**
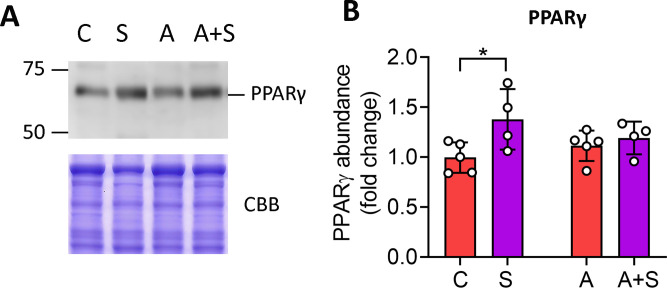
Sucrose but not arsenic exposure promotes PPARγ accumulation in the liver. Western blot analysis of PPARγ protein in the livers of male Wistar rats consuming 20% sucrose (S), 50 ppm sodium arsenite (A) or both (A + S) through drinking water for 2 months. A) Representative Western blot. B) Quantification of PPARγ in the liver samples from 4 animals for each condition. The optical density of the bands was normalized to the optical density of the entire CBB lane. Data are presented as fold change relative to the mean value of the control animals. The bars denote the mean ± SD. Each animal is represented by dots. Analysis by two-way ANOVA with Tukey’s post-hoc test. * denotes statistically significant differences in the post-hoc test with p < 0.05.

Likewise, the livers from S-treated rats displayed higher levels of PPARγ protein compared with C. This effect was attenuated in A + S-treated rats (Fig 5). Thus, inhibition of CAPN1 correlates with PPARγ induction and lipid accumulation in hepatocytes both in vitro and in the liver of S-treated animals but not in A + S.

## 4. Discussion

The signs of metabolic syndrome are closely associated with the development of MASLD, which is the leading cause of hepatic dysfunction worldwide. Both metabolic syndrome and MASLD are caused by the interaction between several endogenous and environmental factors, influencing their pathophysiological mechanisms [2]. We previously showed that sucrose consumption and arsenic exposure promoted the development of metabolic syndrome in rats, interacting in complex ways on hyperinsulinemia, hypertriglyceridemia, and insulin resistance in skeletal muscle [11]. In this work, we deepened the characterization of the effects of sucrose and arsenic exposure for eight weeks on the development of MASLD.

Our results showed that sucrose intake induced hepatic steatosis and fibrosis without altering plasma levels of transaminases at eight weeks of treatment. These data agree with studies in mice, rats, and non-human primates showing that sucrose induces liver steatosis, even if the caloric intake is not increased [25–27].

It is largely recognized that fructose, one of the two monosaccharides composing the disaccharide sucrose, is a potent inductor of plasma triglycerides and MASLD [27]. Mechanistically, fructose metabolism leads to ATP depletion, which promotes the production of uric acid in the liver. This increase in uric acid concentrations induces oxidative stress, reduces fatty acid oxidation, and promotes *de novo* lipogenesis in the liver [27].

Since the molecular mechanisms leading to *de novo* lipogenesis during sub-chronic sucrose consumption remain largely understudied, we focused on alterations of the calpain system. Previous reports show that calpain activity increases in the livers of mice fed a high-fat diet (HFD) for 16 and 18 weeks [9,28]. Also, the protein levels of CAPN1 increased in the livers from non-alcoholic steatohepatitis patients [10]. Hyperhomocysteinemia also induces MASLD associated with increased calpain activity and reduced CAST levels [29]. In contrast, we found that all treatments reduced CAPN1 protein abundance, while sucrose reduced CAST levels in the liver. Likewise, we showed that sucrose and arsenic+sucrose downregulated calpain-dependent proteolysis of αII-spectrin in the liver. Parallelly, we demonstrated that calpain inhibition *in vitro* promoted *de novo* lipogenesis, probably by increasing protein levels of PPARγ in hepatocytes.

Interestingly, arsenic exposure decreased CAPN1 protein abundance but did not alter its proteolytic activity. This apparent contradiction between CAPN1 protein levels and its proteolytic activity is not uncommon. Arsenic impairs calpain activity in lymphocytes *in vitro* without altering the protein levels of CAPN1, CAPN2 and CAPN10, probably due to increased CAST levels and by direct modulation of CAPN1 by arsenic [30]. Moreover, both sucrose and arsenic inhibit the proteolysis of the Tether containing Ubx domain for GLUT4 (TUG) protein carried out by CAPN10 and Usp25m proteases. Nevertheless, the inhibition of TUG proteolysis by arsenic did not correlate with alterations in either CAPN10 nor Usp25m [11]. These discrepancies can be explained by the complex posttranslational regulation that modulates the proteolytic activity of these proteases, such as intracellular Ca2 + levels, changes in subcellular localization, phosphorylation and binding to regulatory proteins [31].

There were several paradoxical effects in animals co-exposed to arsenic + sucrose. While in these animals the levels of hepatic lipids, CAPN1 reduction, and inhibition of αII-spectrin cleavage were similar to those found in animals consuming only sucrose, the levels of PPARγ were not induced in this condition and the extent of fibrosis was reduced compared with those observed in animals consuming sucrose alone. Moreover, in our previous work we observed that arsenic co-exposure prevented the increase in plasma triglycerides induced by sucrose [11]. Studies focused on the interaction between arsenic exposure and consumption of high fat diet showed that depending on the developmental window of exposure and dietary regime, arsenic can increase or lower the effects of the diet on lipid metabolism [32].

On the other hand, the disconnection observed between CAPN1 inhibition, lipid accumulation and PPARγ levels in arsenic+sucrose exposed rats suggest the presence of other calpain-dependent mechanisms involved in sucrose-induced steatosis that are not affected by arsenic and highlight the complex regulation of PPARγ in the context of MASLD. Autophagy participates in the metabolism of lipid droplets in hepatocytes, and its impairment contributes to the development of MASLD [33]. Calpains are known to regulate autophagic flux by cleaving proteins involved in several steps of autophagia [10,34]. Thus, autophagy dysregulation could be an alternative mechanism linking CAPN1 inhibition and hepatic steatosis, which could be insensitive to arsenic.

Additionally, PPARγ levels in the liver can be regulated by the ubiquitin-proteasome system, via ubiquitin-specific protease 2 (USP2). This protease is overexpressed in MASLD patients, where it deubiquitinates PPARγ and favors its transcriptional activity [35]. Whether this mechanism is deregulated by arsenic or sucrose remains to be determined.

Another intriguing finding is the reduction of CAST levels induced by sucrose. In models of MASLD induced by hyperhomocysteinemia, CAST reduction was associated with increased calpain activity, which resulted in the degradation of the inhibitor kappa B alpha (IκBα). This pathway protects the liver from inflammation-induced apoptosis in this model [29]. However, our results showed a decrease in both CAST levels and calpain activity. This discrepancy could be due to the complex prostraslational regulation that controls CAST functions, involving phosphorylation, modulatory proteolysis and subcellular localization [31].

Of note, calpain overactivation in the liver causes oxidative stress, inflammation, necrosis, and lysosome instability, which can be prevented by CAPN1 silencing [10,28]. Nevertheless, CAPN1 KO did not affect liver triglycerides after consuming HFD for 16 weeks [28]. We showed that CAPN1 inhibition promotes PPARγ accumulation and hepatocyte steatosis in vitro. Furthermore, we showed that the effect of CHO on PPARγ levels depend on calpain activity, even in the absence of lipid accumulation. Interestingly, arsenic induced a PPARγ increase in a calpain-dependent fashion, and it had antagonistic effects on PPARγ levels when co-administered with CHO. Since it has been shown that HepG2 cells do not accumulate lipids in response to carbohydrates, we can not confirm whether PPARγ induction by CHO and arsenic is important for lipid accumulation [23,24]. Thus, our results suggest that CAPN1 inhibition in the liver causes *de novo* lipogenesis through PPARγ-dependent and PPARγ-independent mechanisms. Future works in mice models deficient of CAPN1 could demonstrate whether deficiency in this protease increases the susceptibility of developing liver steatosis induced by sucrose, as well as the involved downstream mechanisms.

We propose that both CAPN1 up and downregulation contribute to different aspects of MASLD physiopathology, which seems to depend on the dietary regimen (sucrose vs. high fat diet) as well as the length of the treatment (eight weeks vs more than 16 weeks). Although these results could seem contradictory, similar dual roles of calpain activity have been described in breast cancer, where calpain activity prevents the proliferation of cancer stem cells at the first stages of carcinogenesis while it promotes tumorigenesis and metastasis once the tumor has developed [36,37]. Similarly, both CAPN10 silencing and overexpression cause renal cell death [38,39]. Therefore, the role of calpain deregulation in pathological processes can be highly dependent on the context and the stage of the disease.

In contrast with sucrose intake, arsenic exposure did not cause histological changes in the liver nor affect the development of steatosis, but it reduced the extent of fibrosis induced by sucrose as well as the plasma levels of AST. These findings contrast with works showing that three months of exposure to trivalent arsenicals produces liver steatosis in rats and mice [7,40,41], induces liver fibrosis after six months in mice [42], and high fat-diet intake exacerbates liver fibrosis induced by arsenic exposure after 10 months [43]. These discrepancies with the present work could be due to the length of exposure (eight weeks vs exposures longer than three months) or the administration route (through drinking water vs. gavage). Another plausible explanation could be related to the model organism, as arsenic toxicokinetic differ between rats, mice and humans. For instance, arsenic methylation by hepatocytes is higher in rats than in mice, and arsenic has a high affinity for rat hemoglobin, leading to higher blood accumulation in blood [44,45]. These differences could explain the lack of effect of asenic exposure at eight weeks of treatment. Longer treatments could help further characterize the effects of co-exposure between sucrose and arsenic, as well as the relevance of arsenic-induced CAPN1 alterations in the development of MASLD induced by arsenic. Additionally, since MASLD development depends on sex hormones [12], it remains to be tested whether these responses to arsenic and sucrose differ between females and males.

## 5. Conclusion

We showed that eight weeks of sucrose intake induces liver steatosis and fibrosis, which was associated with impaired CAPN1 activity and increases levels of PPARγ protein. We showed that CAPN1 inhibition can promote both PPARγ accumulation and *de novo* lipogenesis in hepatocytes at least in vitro. In contrast, eight weeks of arsenic exposure did not induce liver steatosis, and although it decreased CAPN1 protein levels, this exposure did not affect its proteolytic activity and PPARγ levels. Interestingly, co-administration of A + S induced steatosis, displayed lower fibrosis levels than sucrose alone, diminished CAPN1 levels but displayed attenuated inhibition of calpain proteolytic activity and did not have increased PPARγ levels.

## Supporting information

S1 Fig Supplementary figure 1. Effects of carbohydrate, arsenic and calpain inhibitor XII in A) cell viability, B) lipid accumulation and C) PPARγ protein levels in HepG2 cells. Data are expressed as the mean ± SD of at least three independent experiments with duplicates. Each dot represents an independent measurement. Data were analyzed by two-way ANOVA with Sydak post hoc test. *p < 0.05 against the C with vehicle.
(TIF)

S2 File Original Western blot images. (PDF)

S3 Table Raw-Data. (XLSX)

S4 Table p values obtained in all ANOVA analyses. (XLSX)

S5 File Publication license of the graphical abstract created using BioRender. (PDF)
