# Supplementary figures and images for: Sucrose but not arsenic induce hepatic steatosis which correlates with calpain-1 inhibition
Source: PLoS One. 2025 Dec 30;20(12):e0339586. doi: 10.1371/journal.pone.0339586 (PMC12752951; doi:10.1371/journal.pone.0339586)

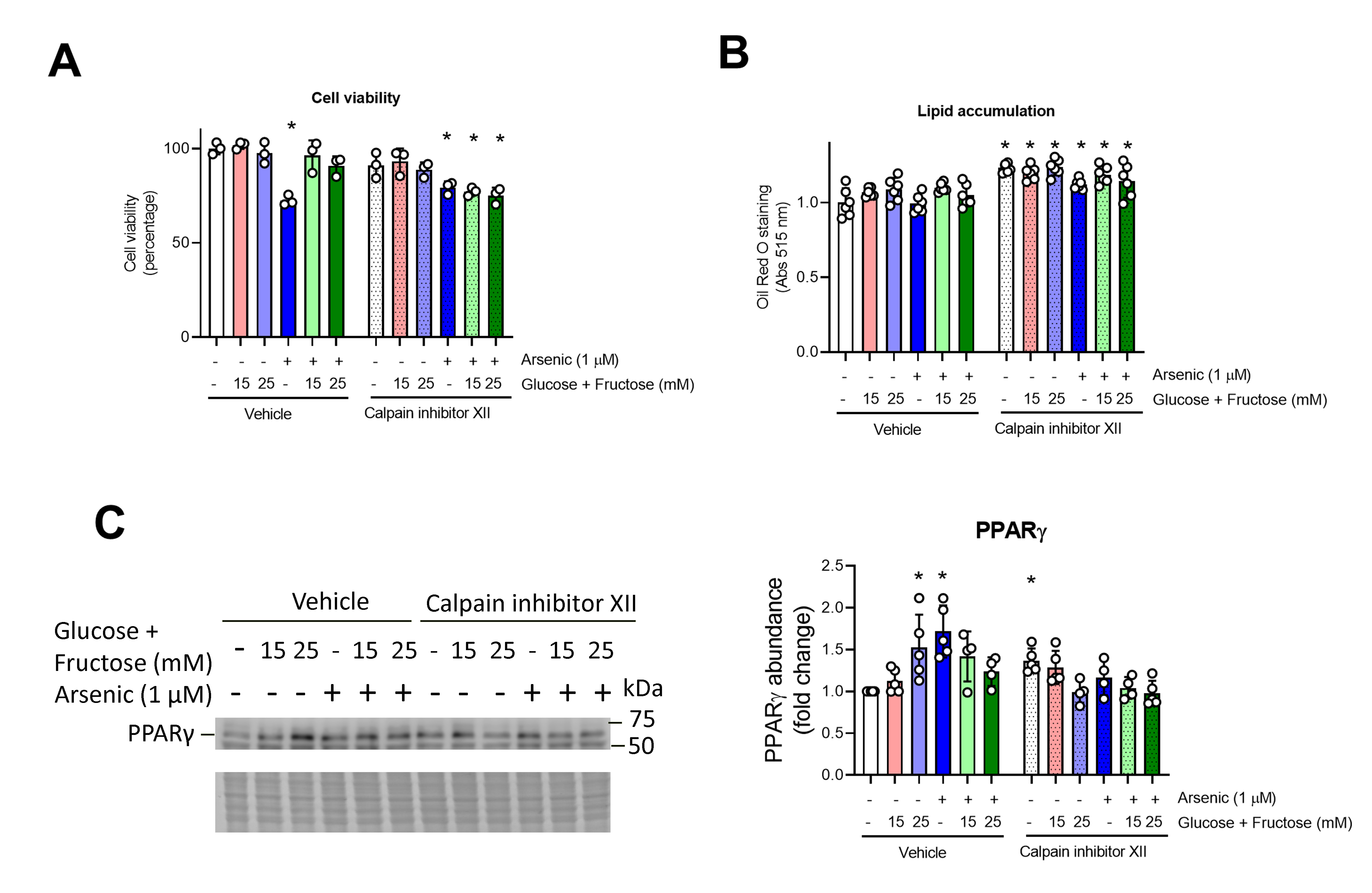

Supplement: S1 Fig — Effects of carbohydrate, arsenic and calpain inhibitor XII in A) cell viability, B) lipid accumulation and C) PPARγ protein levels in HepG2 cells. Data are expressed as the mean ± SD of at least three independent experiments with duplicates. Each dot represents an independent measurement. Data were analyzed by two-way ANOVA with Sydak post hoc test. *p < 0.05 against the C with vehicle. (TIF) [file pone.0339586.s001.tif]
