# Supplementary material for: Sucrose but not arsenic induce hepatic steatosis which correlates with calpain-1 inhibition
Source: PLoS One. 2025 Dec 30;20(12):e0339586. doi: 10.1371/journal.pone.0339586 (PMC12752951; doi:10.1371/journal.pone.0339586)

Original Western blot used for Figure 2

All of the experiments were developed with the ECL prime (Sitiva) reagent in a Li-Cor C-digit scanner (Li-Cor Biosciences) as explained in Methids with the high sensitivity setup. Molecular weight marker was indicated with a dot made with the Westernsure pen (Li-Cor Biosciences).

For the final figure, we used the gel corresponding to samples C2, S2, A2 and AS2 as the representative images.

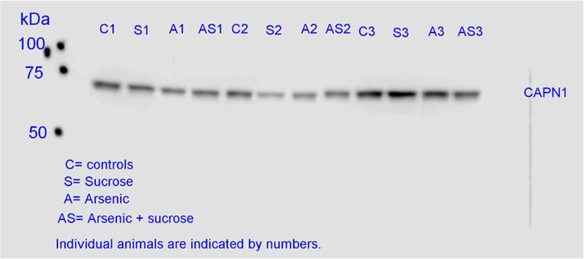

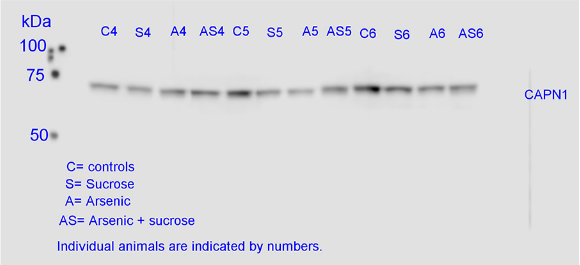

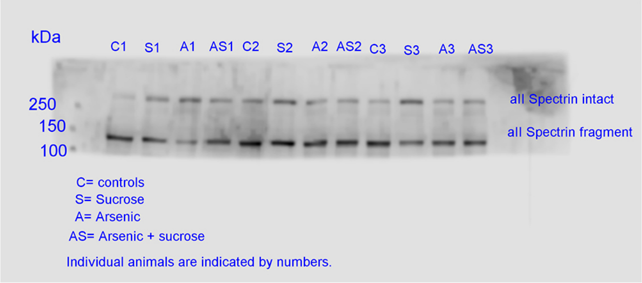

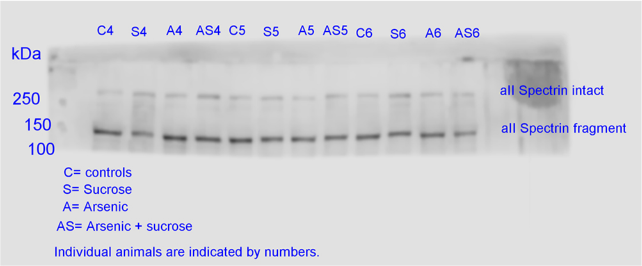

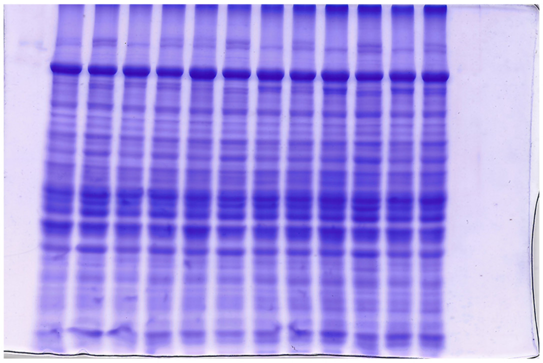

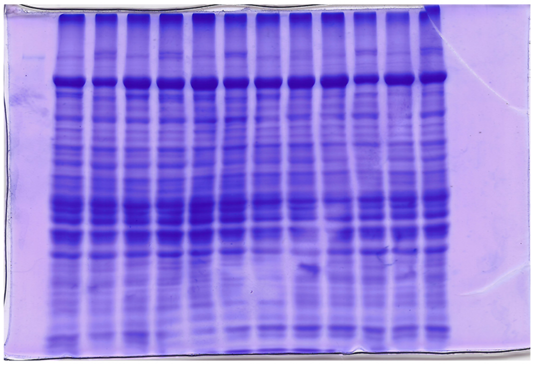

CAPN1 and aII-Spectrin were detected on different sections of the same membrane, so the loading control is the same.

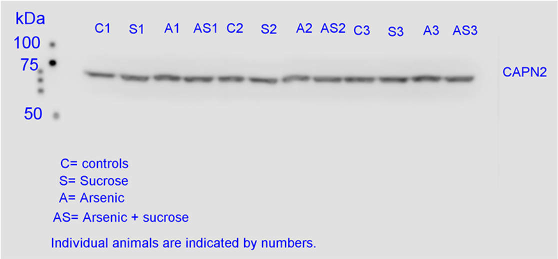

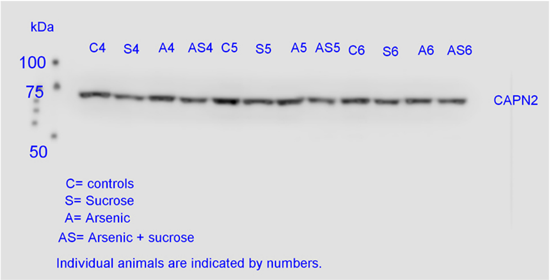

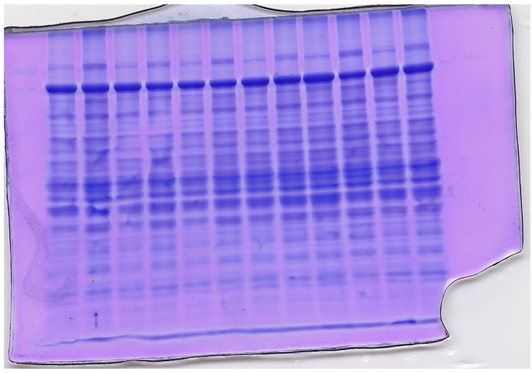

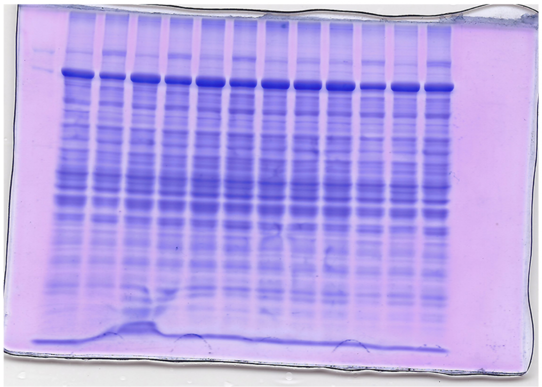

Original Western blot used for Figure 2

All of the experiments were developed with the ECL prime (Sitiva) reagent in a Li-Cor C-digit scanner (Li-Cor Biosciences) as explained in Methids with the high sensitivity setup. Molecular weight marker was indicated with a dot made with the Westernsure pen (Li-Cor Biosciences).

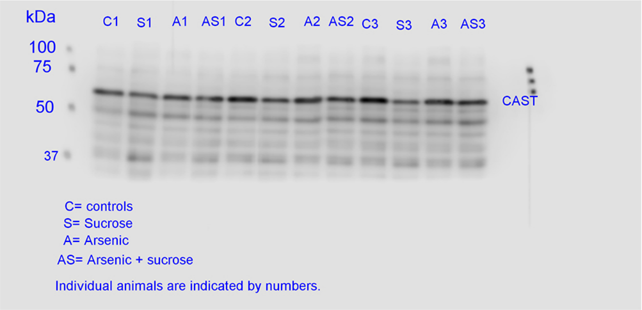

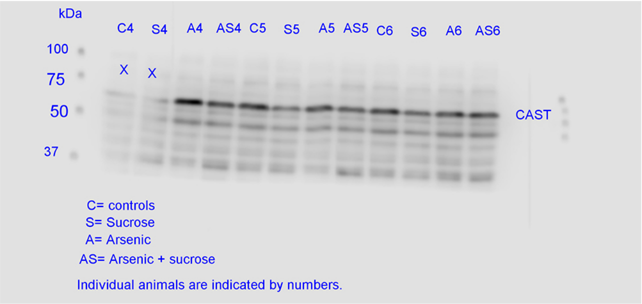

X: samples excluded from the final analysis

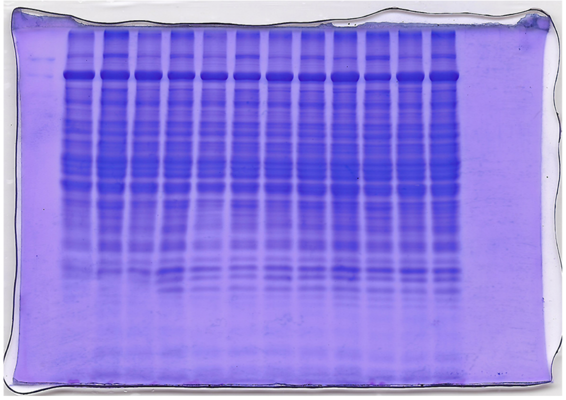

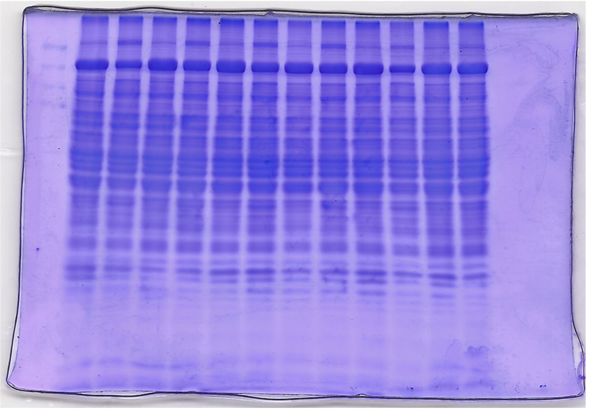

Original Western blot used for Figure 4

Independent biological experiments are denoted as Experiment#. For the final figure, we selected the Experiment 1

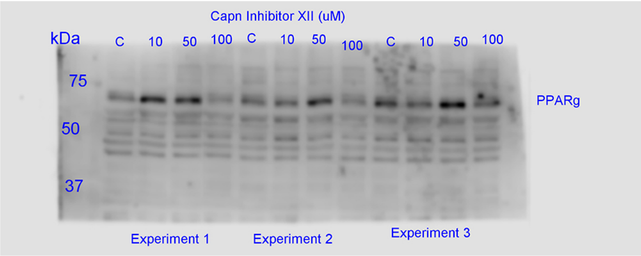

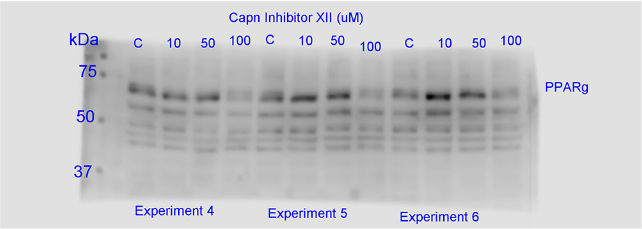

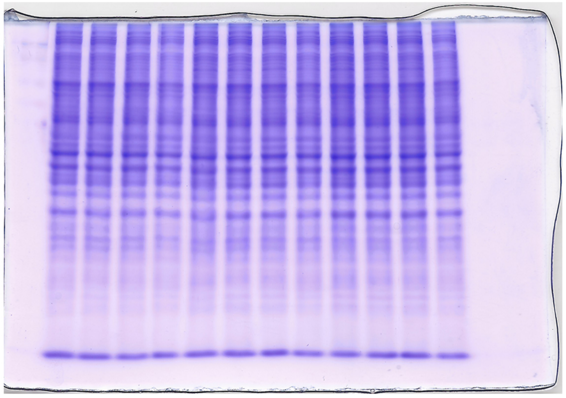

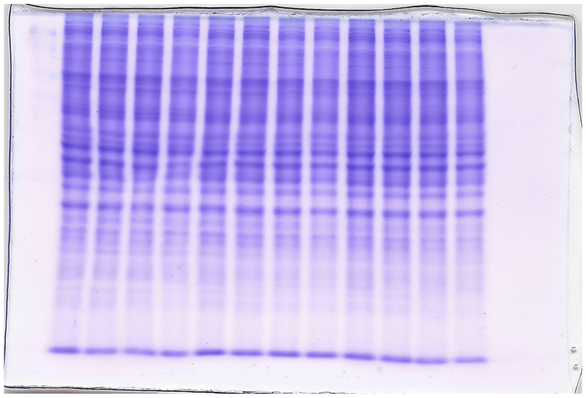

Original Western blot used for Figure 5

All of the experiments were developed with the ECL prime (Sitiva) reagent in a Li-Cor C-digit scanner (Li-Cor Biosciences) as explained in Methids with the high sensitivity setup. Molecular weight marker was indicated with a dot made with the Westernsure pen (Li-Cor Biosciences). The gel used as a representative Western blot for figure 5 corresponds to samples C4, S4, A4 and As4

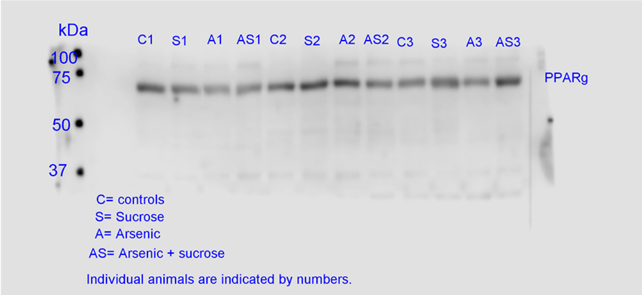

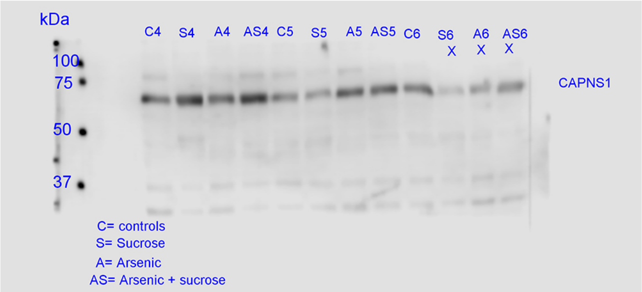

X: samples excluded from the final analysis

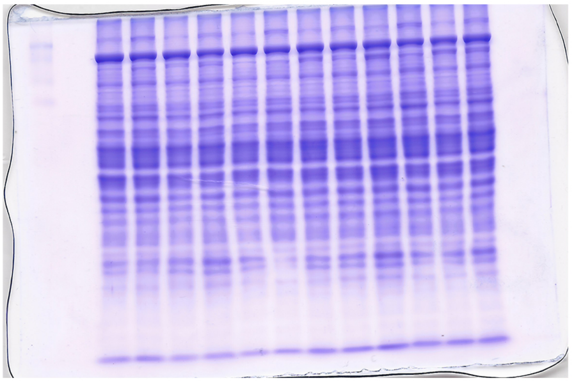

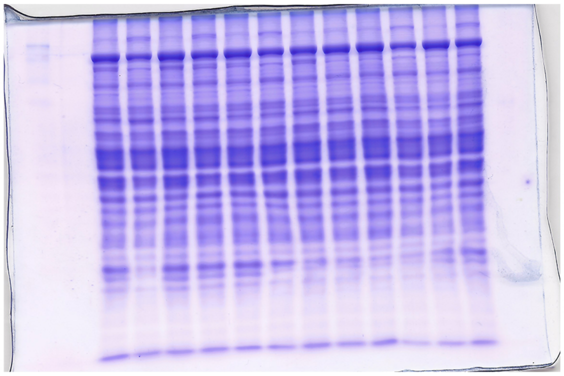

Supplement: S2 File — (PDF) [file pone.0339586.s002.pdf]
